# Empowering Rural Dementia Caregivers Through a Technology-based Platform: A Case Study from Michigan

**DOI:** 10.1093/geroni/igaf122.3924

**Published:** 2025-12-31

**Authors:** Lora Chehab, Rhea Shenoy, Fei Sun, Daniel Velez Ortiz

**Affiliations:** Michigan State University, East Lansing, Michigan, United States; Michigan State University, East Lansing, Michigan, United States; Michigan State University, East Lansing, Michigan, United States; Michigan State University, East Lansing, Michigan, United States

## Abstract

Providing care for relatives with dementia poses additional challenges for families in rural communities where access to information, education, and supportive services is often limited. This case study examines the implementation of a technology-based platform, Trualta, to support dementia caregivers in 12 selected rural counties in Michigan by providing education, skills training, peer connection, and local resources (e.g., adult day centers, county specific resource guides). Once logged in, caregivers have on demand access to the platform based upon their needs. Data from 172 caregivers enrolled between July 2023 to July 2025 were analyzed to assess usage patterns, impact, and lessons learned. On average, caregivers completed 13.5 learning modules (SD = 32.37), which are dementia care education and skills training; 41 participants attended at least one synchronous seminar. Participants viewed local resources an average of 4.2 times (SD = 5.0). Trualta’s peer support forum provides caregivers with space to share questions, reflections, and experiences. On average, participants contributed 2.74 entries to the forum (SD = 10.40), reflecting meaningful engagement. A thematic analysis of posts revealed that the two most prominent categories were daily care techniques and emotional support, which accounted for 33.0% and 17.4% of all entries, respectively. Interaction analysis found posts were notably detailed and insightful, with an average length of 633 characters and 58.0% using structured titles. Findings indicate technology-based interventions can empower rural dementia caregivers by addressing their needs for education, emotional support, and community resources. However, future research needs to evaluate the program’s long-term impact on caregiver stress and well-being.

